# A23 ESTIMATING INDIRECT AND OUT-OF-POCKET COSTS IN PEDIATRIC INFLAMMATORY BOWEL DISEASE: A NATION-WIDE CROSS-SECTIONAL ANALYSIS

**DOI:** 10.1093/jcag/gwab049.022

**Published:** 2022-02-21

**Authors:** W El-Matary, J Witt, C N Bernstein, K Jacobson, D R Mack, A Otley, T Walters, H Q Huynh, J deBruyn, A Griffiths, E I Benchimol

**Affiliations:** 1 Pediatric Gastroenterology, University of Manitoba Faculty of Health Sciences, Winnipeg, MB, Canada; 2 University of Manitoba, Winnipeg, MB, Canada; 4 BC Children’s Hospital, Vancouver, BC, Canada; 5 University of Ottawa, Ottawa, ON, Canada; 6 Dalhousie University, Halifax, NS, Canada; 7 The Hospital for Sick Children, Toronto, ON, Canada; 8 Pediatrics, University of alberta, Edmonton, AB, Canada; 9 University of Calgary, Calgary, AB, Canada; 10 Hospital for Sick Children, Toronto, ON, Canada; 11 Gastroenterology, Hepatology and Nutrition, The Hospital for Sick Children Department of Paediatrics, Toronto, ON, Canada

## Abstract

**Background:**

Identifying disease-related costs is a crucial step to plan for proper allocation of resources and future healthcare services for persons with inflammatory bowel disease (IBD). Data on pediatric inflammatory bowel disease-associated costs are limited.

**Aims:**

We aimed to estimate indirect and out of pocket (OOP) pediatric IBD-associated costs in Canada.

**Methods:**

In a nation-wide cross-sectional analysis, caregivers of children and young adults (<17 years) with IBD were invited to complete a questionnaire on lost work hours and OOP costs related to IBD in the 4 weeks prior to the survey. Participants were re-invited to periodically answer the same questionnaire every 3–9 months for 2 years. Lost productivity was calculated using the Human Capital method. Costs were reported in 2018 inflation-adjusted Canadian dollars. Predictors of high cost users (top 25%) were examined using negative binomial regression.

**Results:**

Consecutive 243 (82 incident cases) of 262 (92.7%) approached participants completed the first survey with a total of 450 surveys longitudinally completed over 2 years. The annual median indirect costs per patient were $5,951 (IQR $1,812- $12,278), with $5,776 (IQR $1,465-$11,733) for Crohn’s disease (CD) and $6,084 (IQR $2,470-$13,371) for ulcerative colitis (UC) (p=0.77). The annual median per patient OOP costs were $2,925 (IQR $978- $8,125) with $3,021 (IQR $978- $8,125) for CD and $2,600 (IQR $975- $8,125) for UC (p=0.55). Older age (10-17y) at diagnosis (p=0.04) and parents in part-time employment (p=0.01) were predictors of higher indirect costs, while female sex (p<0.001), parents with a lower education level (p<0.001) and lower annual family income (p<0.01) were associated with higher OOP costs.

**Conclusions:**

Indirect and OOP IBD-associated costs are substantial and more likely to affect families with unstable employment and lower annual income. Examining different strategies and interventions to reduce these costs such as virtual platforms, telephone and outreach clinics especially in poor communities and families with low annual income is warranted.

**Funding Agencies:**

CIHRThe Children’s Hospital Research Institute of Manitoba

